# Supplementary material for: Cell-specific CRISPR–Cas9 activation by microRNA-dependent expression of anti-CRISPR proteins
Source: Nucleic Acids Res. 2019 Apr 15;47(13):e75. doi: 10.1093/nar/gkz271 (PMC6648350; doi:10.1093/nar/gkz271)
Supplement: gkz271_Supplemental_Files [file gkz271_supplemental_files.zip › Cas-ON_Hoffmann_et_al_supplements_production.pdf]

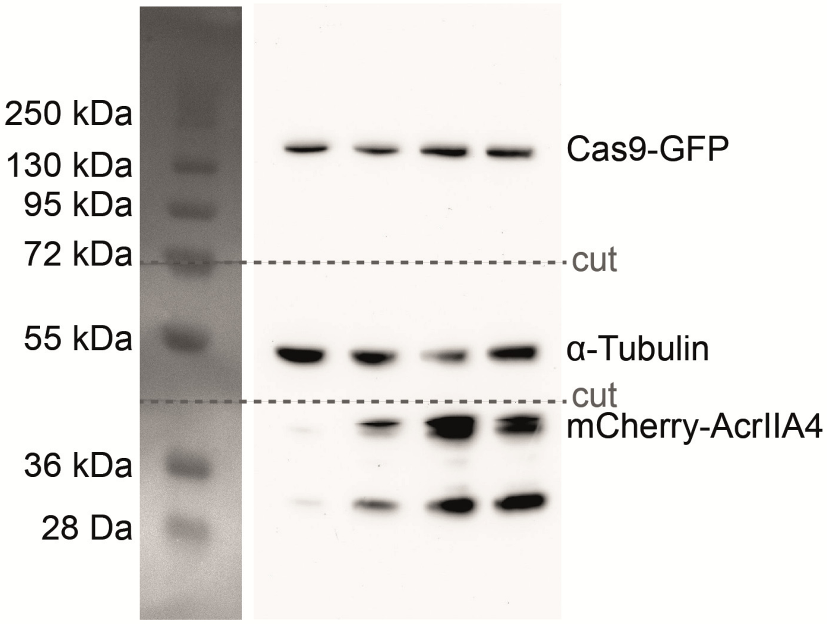

Supplementary Figure S1. Full-length Western blot image (corresponds to Figure 1D). The ladder is the PageRuler Prestained Protein Ladder (ThermoFisher). Positions at which the membrane was cut prior to antibody incubation are indicated (dashed lines).

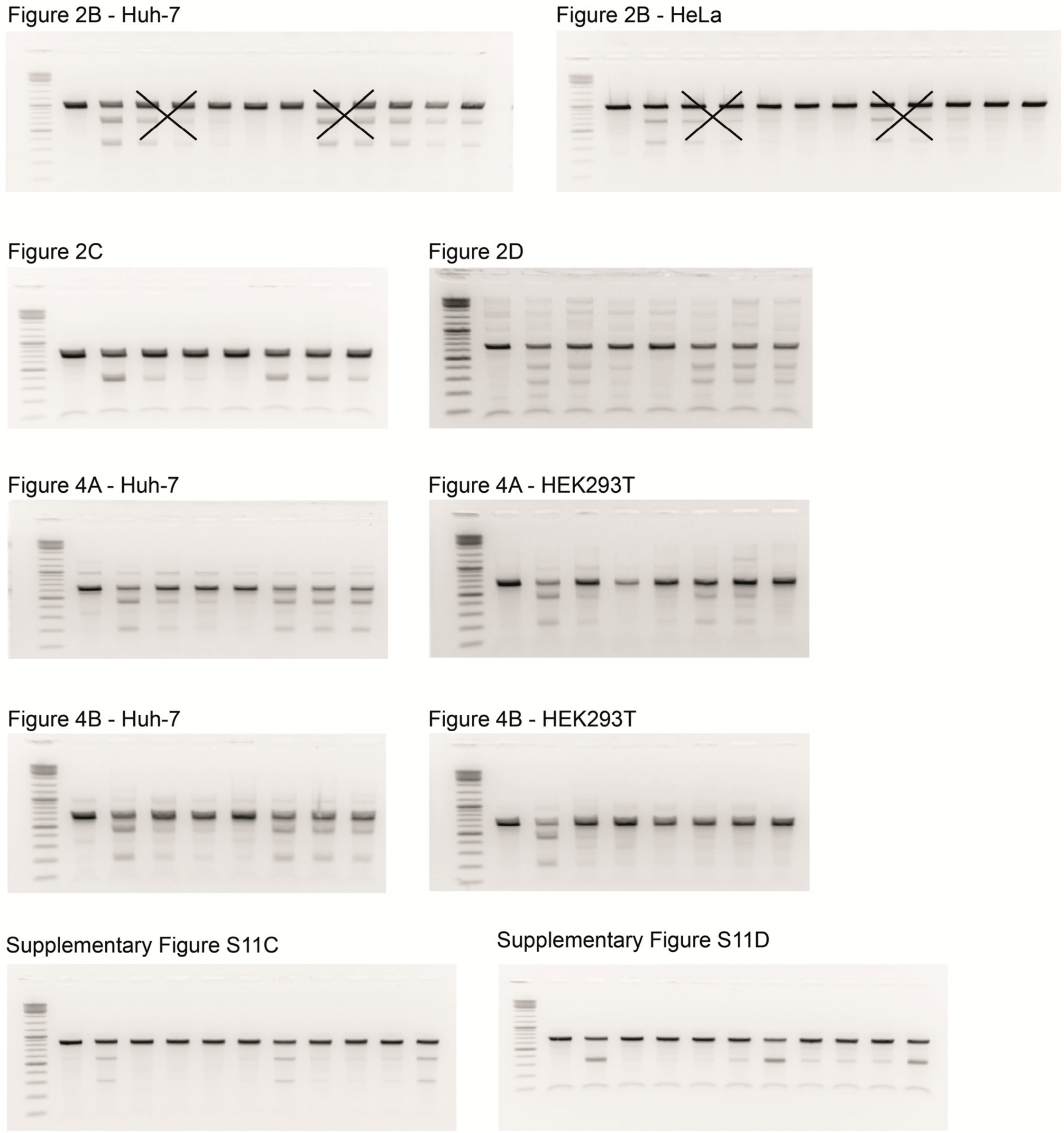

Supplementary Figure S2. Full-length gel images of T7 endonuclease assays. The ladder is the Gene Ruler DNA Ladder Mix (Thermo Fisher).

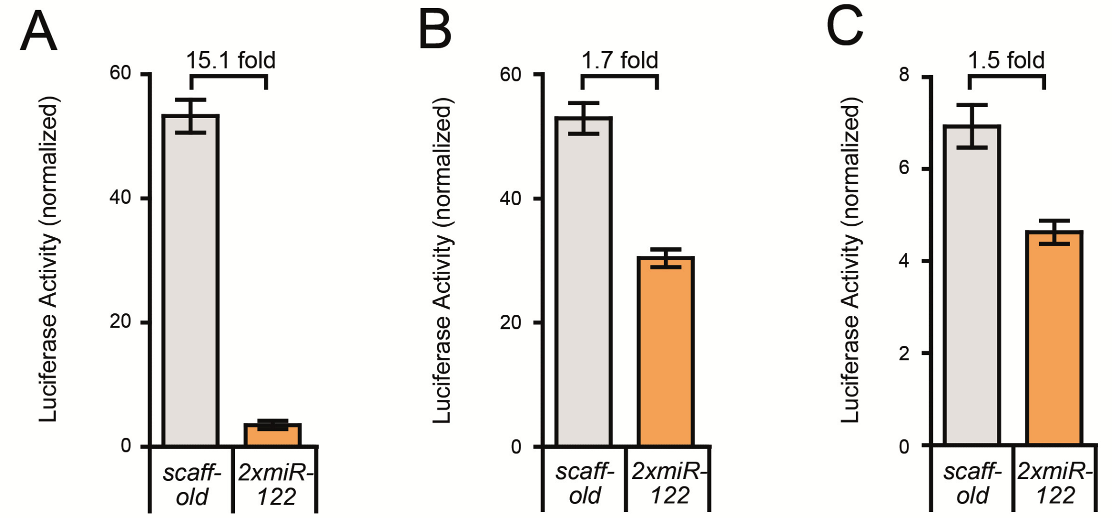

Supplementary Figure S3. MiRNA-122 is highly abundant specifically in hepatocytes. Huh-7 (**A**), HeLa (**B**), and HEK293T (**C**) cells were transfected with a luciferase reporter construct carrying two miR-122 binding sites in the 3'UTR (2xmiR-122) or not (scaffold, as control), followed by luciferase assay. The reporter bearing the miR-122 binding sites is efficiently knocked down in Huh-7 cells, but only mildly affected in HeLa or HEK293T cells. Data are means ± s.e.m. (n = 3 independent experiments).

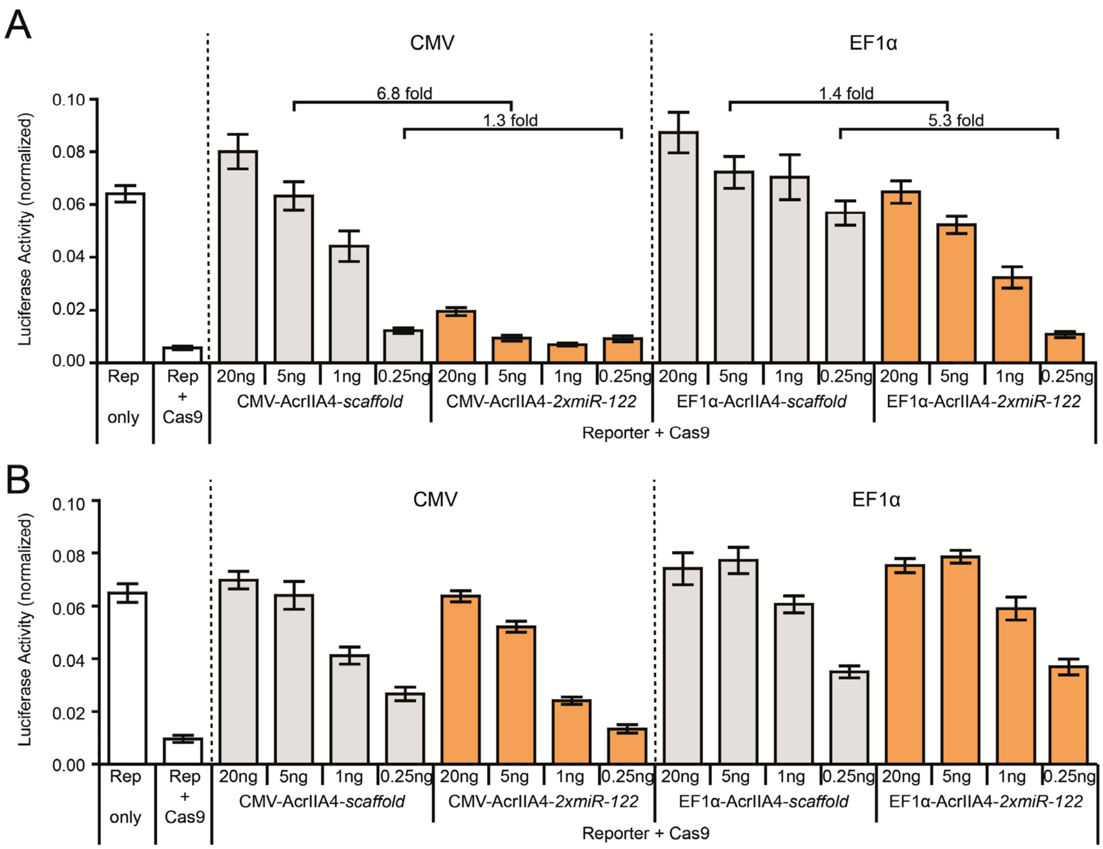

Supplementary Figure S4. *Spy*Cas9 inhibition can be modulated by tuning the strength of the AcrIIA4-driving promoter. Huh-7 cells (**A**) and HeLa (**B**) cells were co-transfected with constructs encoding *Spy*Cas9, a luciferase reporter, a reporter-targeting sgRNA, and AcrIIA4-*2xmiR-122* or AcrIIA4-*scaffold* as control, followed by luciferase assay. A CMV promoter or an EF1α promoter was used to drive Acr expression and the Acr vector dose was varied during transfection as indicated. Data are means ± s.e.m. (n = 3 independent experiments).

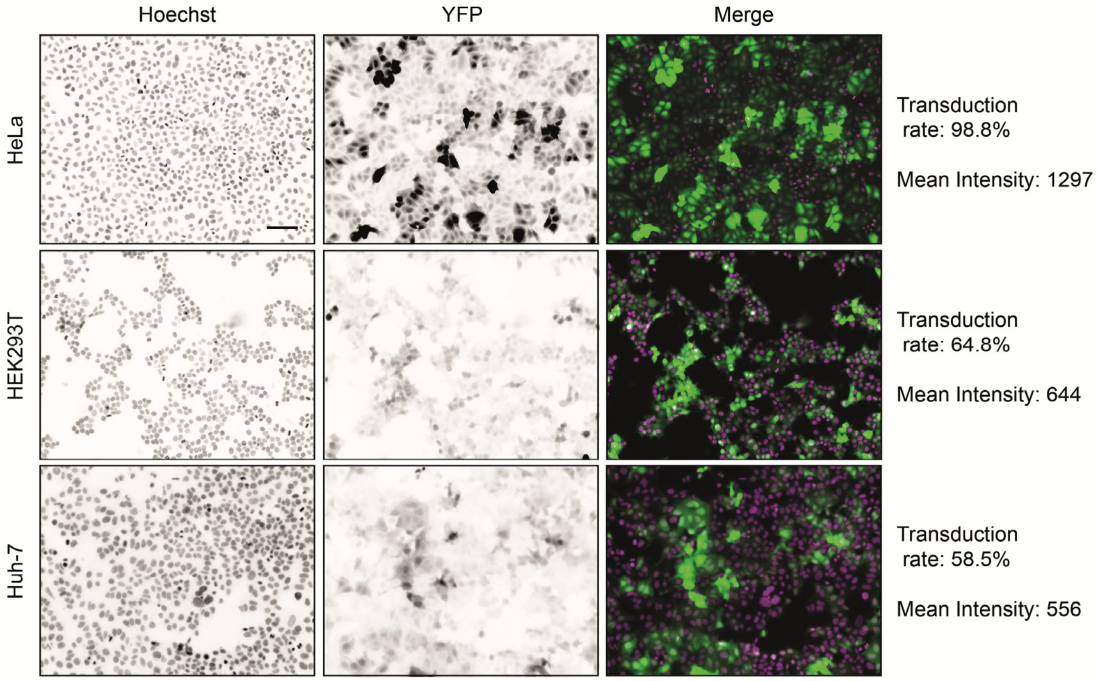

Supplementary Figure S5. AAV2 efficiently transduces different cell lines. Representative fluorescence microscopy images of HeLa, HEK293T and Huh-7 cells infected with AAV2 encoding a YFP reporter. Scale bar 100 µm. Automated image analysis was used to quantify the number of YFP-positive cells (transduction rate) as well as the mean intensity of YFP fluorescence (see Material and Methods for details).

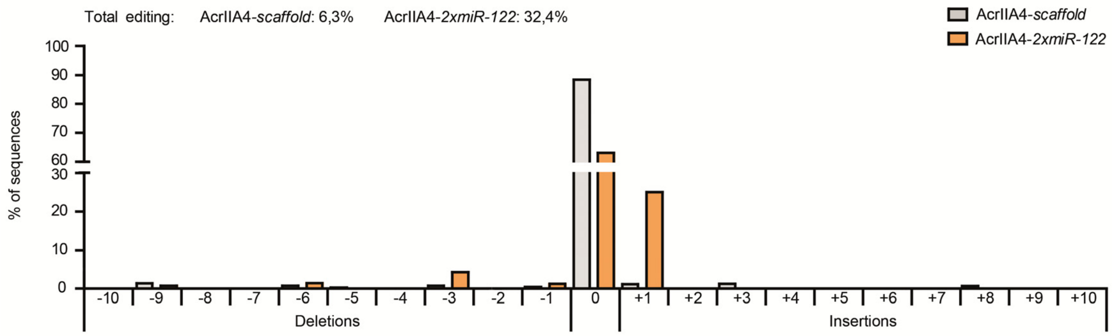

Supplementary Figure S6. MiR-122-dependent editing of the EMX1 locus in hepatocytes. Huh-7 cells were co-transduced with AAV vectors expressing *Spy*Cas9, a sgRNA targeting the human EMX1 locus, and AcrIIA4-*2xmiR-122* or AcrIIA4-*scaffold* (as control). TIDE sequencing revealed a high frequency of insertions and deletions in the AcrIIA4-*2xmiR-122*, but not in the AcrIIA4-*scaffold* sample. The total editing efficiencies as calculated by the TIDE algorithm are indicated on top. Data for a representative sample is shown.

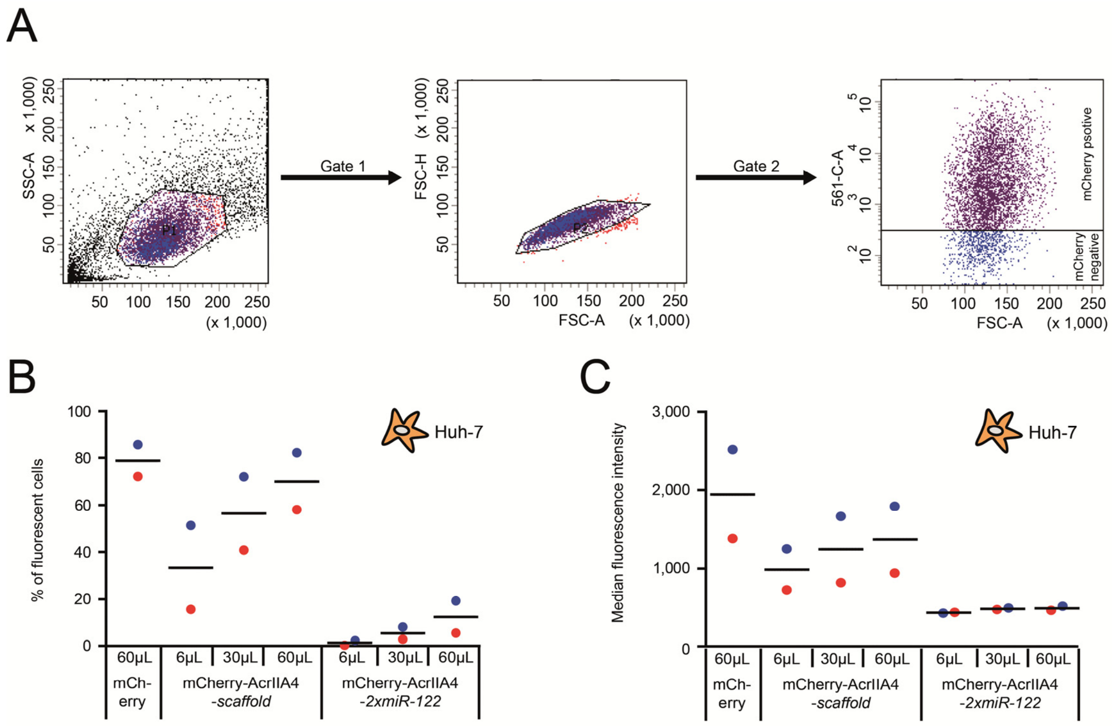

Supplementary Figure S7. FACS analysis of AcrIIA4 expression in Huh-7 cells upon AAV delivery. Cells were transduced with 6, 30 or 60 µL of AAV vectors encoding either mCherry-AcrIIA4-*scaffold* or mCherry-AcrIIA4-2x*miR-122*, followed by flow cytometry. (**A**) Gating strategy to obtain mCherry-positive fraction. The exemplary plots show data for the mCherry positive control. (**B,C**) Percentage of mCherry-positive cells (**B**) and median mCherry fluorescence intensity (**C**) of the indicated samples. Data are means (black bars) from two independent experiments (blue dots: replicate 1; red dots: replicate 2).

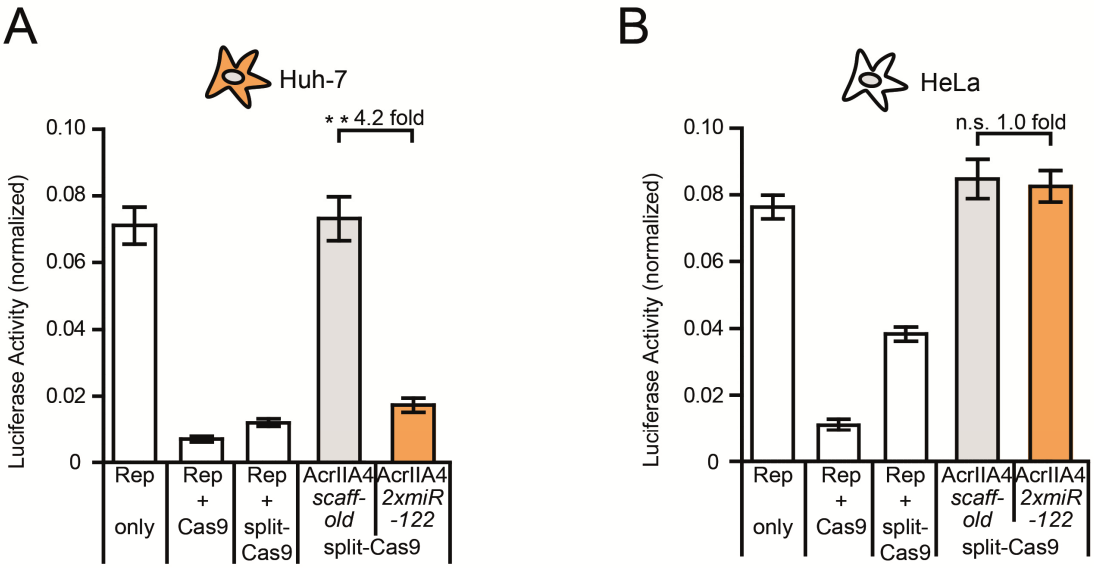

Supplementary Figure S8. Hepatocyte-specific split-*Spy*Cas9 activity. Huh-7 (**A**) and HeLa (**B**) cells were co-transfected with constructs expressing a luciferase reporter, a reporter-targeting sgRNA, an N- and C-terminal *Spy*Cas9 fragment fused to split-inteins, and AcrIIA4-*2xmiR-122* or AcrIIA4-*scaffold* (as control), followed by luciferase assay. Data are means ± s.e.m. (n = 4 independent experiments). n.s. = not significant, **P < 0.01, by two-sided Student's *t*-test with Bonferroni correction. Precise *P*-values are shown in Table 1 (Material and Methods).

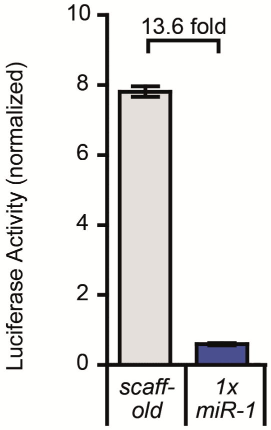

Supplementary Figure S9. MiRNA-1-mediated reporter knockdown in murine cardiomyocytes. HL-1 cells were transduced with AAVs encoding a luciferase reporter construct carrying a miR-1 target site in the 3'UTR (1xmiR-1) or not (scaffold, as control), followed by luciferase assay. Data are means ± s.e.m. (n = 3 replicates, i.e. parallel transductions).

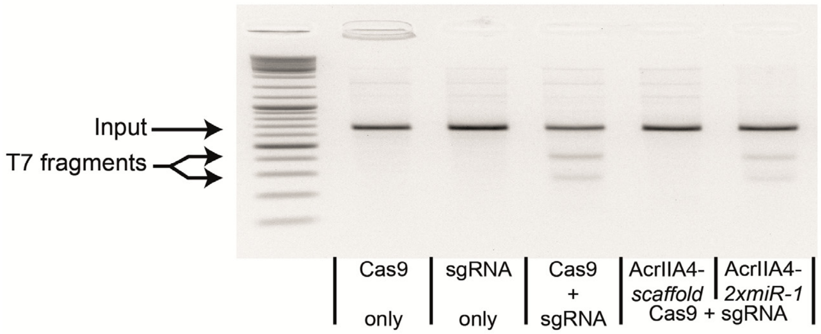

Supplementary Figure S10. MiR-1-dependent gene editing in cardiomyocytes. HL-1 cells were co-transduced with AAV vectors encoding *Spy*Cas9, a sgRNA targeting the Rosa-26 locus, and either AcrIIA4-*2xmiR-1* or AcrIIA4-*scaffold* (control), followed by T7 endonuclease assay. Data for a representative sample is shown.

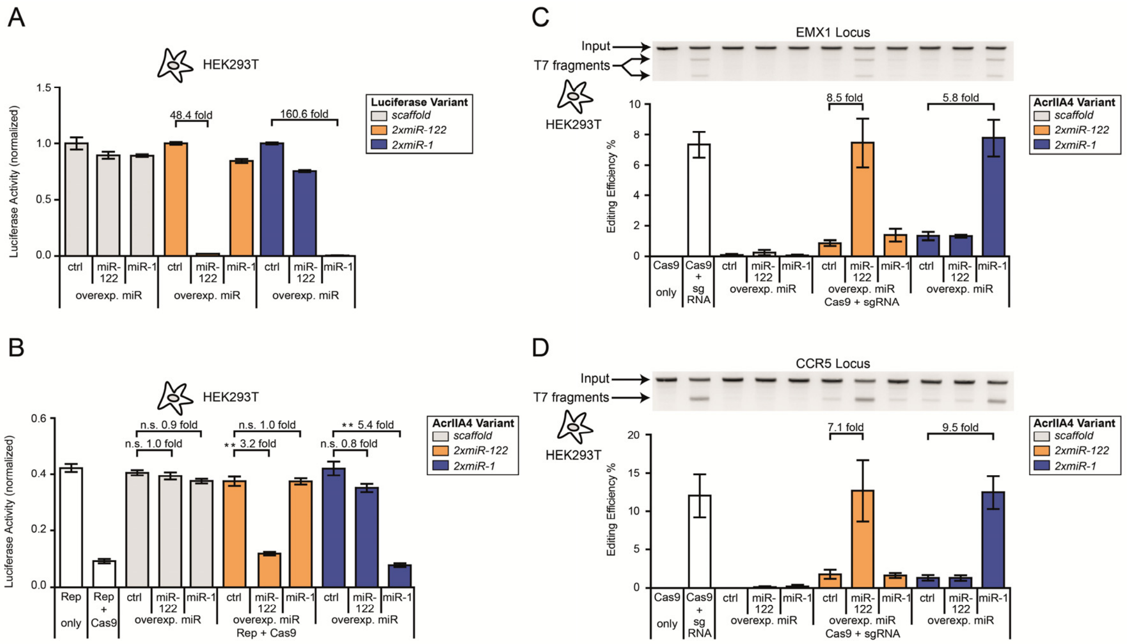

Supplementary Figure S11. The miR-122- and miR-1-dependent Cas-ON switches are orthogonal. (**A**) HEK293T cells were transfected with (i) a luciferase reporter construct carrying two miR-122, two expressing miR-122, miR-1 or an empty vector, followed by luciferase assay. Data are means ± s.e.m. (n = 3 replicates, i.e. parallel transfections). (**B**) MiRNA-dependent luciferase reporter cleavage by Cas9. HEK293T cells were co-transfected with plasmids encoding (i) *Spy*Cas9, (ii) a luciferase reporter and a reporter-targeting sgRNA, (iii) AcrIIA4-*2xmiR-122*, AcrIIA4-*2xmiR-1* or AcrIIA4-*scaffold* (as control), and (iv) miR-122 or miR-1 overexpression vectors or an empty vector, followed by luciferase assay. Data are means ± s.e.m. (n = 3 independent experiments). $^{**}P < 0.01$, by two-sided Student's *t*-test with Bonferroni correction. Precise *P*-values are shown in Table 1 (Material and Methods). (**C, D**) MiRNA-dependent editing of endogenous loci. HEK293T cells were co-transduced with AAV vectors encoding (i) *Spy*Cas9, (ii) a sgRNA targeting the human EMX1 (**C**) or CCR5 (**D**) locus, (iii) AcrIIA4-*scaffold*, AcrIIA4-2x*miR-122* or AcrIIA4-2x*miR-1*, and (iv) miR-122 or miR-1 expression vectors or an empty vector, followed by T7 endonuclease assay. Data are means ± s.e.m. (n = 3 independent experiments). (**A-D**) ctrl, control. overexp., overexpressed.

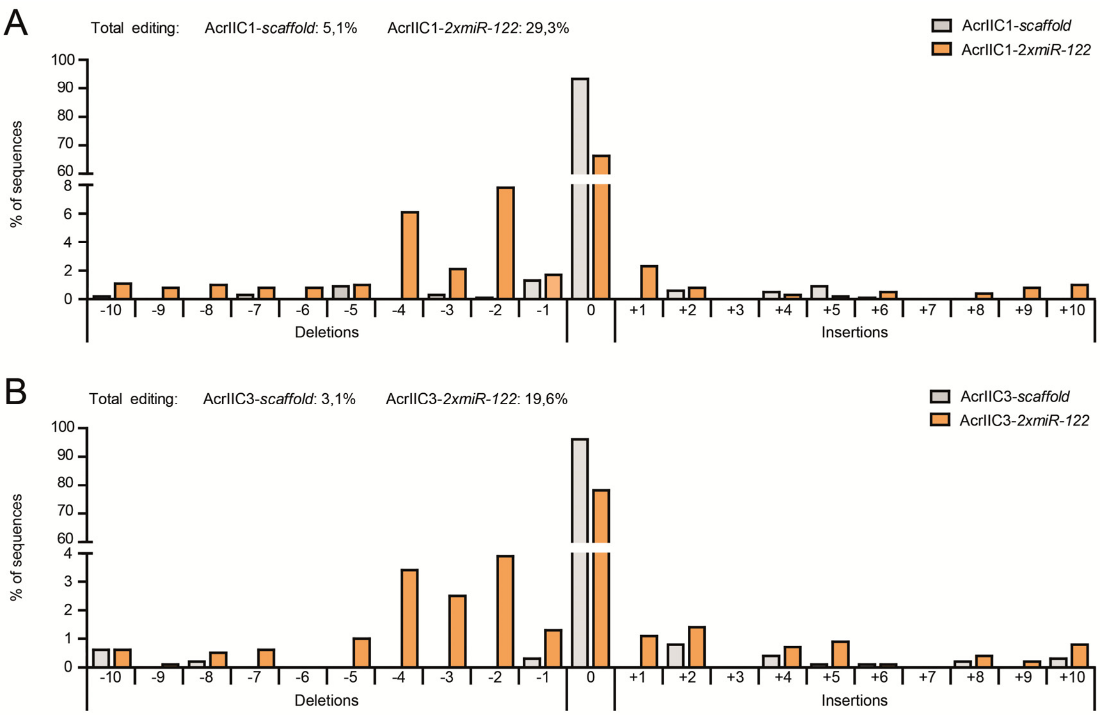

Supplementary Figure S12. MiR-122-dependent genome editing by *Nme*Cas9. Huh-7 cells were co-transduced with AAV vectors encoding *Nme*Cas9, a sgRNA targeting the human VEGFA locus, and either AcrIIC1-*2xmiR-122* or AcrIIC1-*scaffold* (**A**), or AcrIIC3-*2xmiR-122* or AcrIIC3-*scaffold* (**B**). TIDE sequencing revealed a broad range of insertions and deletions for the AcrIIC1-*2xmiR-122*/AcrIIC3-*2xmiR-122* samples, but not for the AcrIIC1-*scaffold*/AcrIIC3-*scaffold* control samples. The total editing efficiencies as calculated by the TIDE algorithm are indicated on top. Data for a representative samples are shown.

Supplementary Table S1. List of constructs created and used in this study. ITR, inverted terminal repeat. TK, thymidine kinase. SV40, simian virus 40. BGH, bovine growth hormone. LTR, long terminal repeat. CMV, cytomegalovirus. RSV, respiratory syncytial virus. EF1α, elongation factor 1α

No	Name/Group	Insert description
	**Reporter**	
**1**	Luciferase cleavage reporter (to measure *Spy*Cas9 activity)	3'ITR, SV40 promoter, *Renilla luciferase*, TK promoter, Firefly luciferase, SV40 polyA, H1 promoter, sgRNA(Firefly luciferase), 5' ITR
**2**	Tet-inducible luciferase reporter (Addgene # 64127, to measure *Spy*dCas9-VP64 activity)	Tet-responsive elements, ECFP, CMV promoter, Firefly luciferase, SV40 polyA
**3**	pRL-TK (Promega)	TK promoter, *Renilla* luciferase, SV40 polyA
**4**	pSiCheck-2 (no miR target site, control)	SV40 promoter, *Renilla* luciferase, scaffold, BGH polyA, TK promoter, Firefly luciferase
**5**	pSiCheck-2 (2xmiR-122 target sites)	SV40 promoter, *Renilla* luciferase, 2xmiR-122 binding sites, BGH polyA, TK promoter, Firefly luciferase
**6**	pSiCheck-2 (2xmiR-1 target sites)	SV40 promoter, *Renilla* luciferase, 2xmiR-1 binding sites, BGH polyA, TK promoter, Firefly luciferase
**7**	pAAV-pSi (no miR target site control)	3'ITR, SV40 promoter, *Renilla* luciferase, TK promoter, Firefly luciferase, SV40 promoter, 5'ITR
**8**	pAAV-pSi (1xmiR-1 target site)	3'ITR, SV40 promoter, *Renilla* luciferase, 1xmiR-1 binding site, TK promoter, Firefly luciferase, SV40 polyA, 5'ITR
**9**	scAAV-YFP (YFP reporter)	AAV4-ITR, CMV promoter, YFP, BGH polyA, AAV2-ITR
	**Cas9 Variants**	
**10**	CMV-*Spy*Cas9 (Addgene # 113033)	CMV promoter, NLS-*Spy*Cas9-NLS, BGH polyA
**11**	*Spy*Cas9-GFP	CMV promoter, NLS-*Spy*Cas9-NLS-GFP, BGH polyA
**12**	dCAS9-VP64_GFP (Addgene # 61422)	CMV promoter, 5'LTR(HIV-1), EF1α promoter, NLS-*Spy*dCas9-NLS-VP64(Transactivator)-T2A-EGFP, 3'LTR(HIV-1)
**13**	pAAV-CMV-*Spy*Cas9 (Addgene # 113034)	3'ITR, minimal CMV promoter, NLS-*Spy*Cas9-NLS, 5'ITR
**14**	AAV N-*Spy*Cas9-Intein, U6-sgRNA scaffold (F+E) (Addgene # 120293)	3'ITR, SMVP promoter (Reference 1), NLS-*Spy*Cas9(N)-N-intein, SV40 polyA, U6, *Spy*Cas9 sgRNA scaffold(F+E), 5'ITR
**15**	AAV Intein-C-*Spy*Cas9	3'ITR, SMVP promoter, C-intein-*Spy*Cas9(C), SV40 polyA, XbaI site, MluI site, 5'ITR
**16**	AAV Intein-C-*Spy*Cas9, CMV-AcrIIA4-*scaffold* (2xBsmBI sites) (Addgene # 120294)	3'ITR, SMVP promoter, C-intein-*Spy*Cas9(C), SV40 polyA, CMV promoter, AcrIIA4, scaffold sequence, BGH polyA, 5'ITR
**17**	AAV Intein-C-*Spy*Cas9, CMV-AcrIIA4-*2xmiR-122* target sites (Addgene # 120295)	3'ITR, SMVP promoter, C-intein-*Spy*Cas9(C), SV40 polyA, CMV promoter, AcrIIA4, 2xmiR-122 binding sites, BGH polyA, 5'ITR

Supplementary Table S1 continued

**18**	pEJS654 All-in-One AAV-sgRNA-h*Nme*Cas9 (Addgene # 112139)	AAV2-ITR, *Nme*Cas9 sgRNA scaffold, U6 promoter, U1a promoter, NLS-*Nme*Cas9-NLSs, β-globin polyA, AAV2-ITR
**19**	All-in-One AAV-sgRNA-VEGFA-h*Nme*Cas9	AAV2-ITR, *Nme*Cas9-sgRNA(VEGFA), U6 promoter, U1a promoter, NLS-*Nme*Cas9-NLSs, β-globin polyA, AAV2-ITR

sgRNAs

**20**	sgRNA1_Tet-inducible Luciferase reporter (Addgene # 64161)	U6 promoter, *Spy*Cas9-sgRNA(Tet-inducible promoter)
**21**	pAAV-RSV-GFP-U6-EMX1 sgRNA (*Spy*Cas9 scaffold) (Addgene # 113040)	AAV2-ITR, RSV promoter, EGFP, U6 promoter, *Spy*Cas9-sgRNA(EMX1), AAV4-ITR
**22**	pAAV-RSV-GFP-U6-Rosa-26 sgRNA (*Spy*Cas9 scaffold) (Addgene # 120296)	AAV2-ITR, RSV promoter, EGFP, U6 promoter, *Spy*Cas9-sgRNA(Rosa-26), AAV4-ITR
**23**	pAAV-RSV-GFP-U6-CCR5 sgRNA (*Spy*Cas9 scaffold) (Addgene # 113041)	AAV2-ITR, RSV promoter, EGFP, U6 promoter, *Spy*Cas9-sgRNA(CCR5), AAV4-ITR
**24**	pAAV-RSV-GFP-U6-AAVS1 sgRNA (*Spy*Cas9 scaffold)	AAV2-ITR, RSV promoter, EGFP, U6 promoter, *Spy*Cas9-sgRNA(AAVS1), AAV4-ITR

Anti-CRISPR Variants

**25**	CMV-mCherry-AcrIIA4-*scaffold*	CMV promoter, NLS-mCherry-AcrIIA4, scaffold sequence, BGH polyA
**26**	CMV-mCherry-AcrIIA4 –2x*miR-122*	CMV promoter, NLS-mCherry-AcrIIA4, 2xmiR-122 binding sites, BGH polyA
**27**	CMV-AcrIIA4-*scaffold*	CMV promoter, AcrIIA4, scaffold sequence, BGH polyA
**28**	CMV-AcrIIA4-2x*miR-122*	CMV promoter, AcrIIA4, 2xmiR-122 binding sites, BGH polyA
**29**	CMV-AcrIIA4-2x*miR-1*	CMV promoter, AcrIIA4, 2xmiR-1 binding sites, BGH polyA
**30**	AAV EF1α-AcrIIA4-*scaffold*	AAV4-ITR, H1 promoter, *Spy*Cas9-sgRNA-scaffold, EF1α promoter, AcrIIA4, scaffold sequence, BGH polyA, AAV2-ITR
**31**	AAV EF1α- AcrIIA4-2x*miR-122*	AAV4-ITR, H1 promoter, *Spy*Cas9-sgRNA-scaffold, EF1α promoter, AcrIIA4, 2xmiR-122 binding sites, BGH polyA, AAV2-ITR
**32**	AAV CMV-driven AcrIIA4-*scaffold* (Addgene # 120297)	AAV4-ITR, U6 promoter, *Spy*Cas9-sgRNA-scaffold, CMV promoter, AcrIIA4, scaffold sequence, BGH polyA, AAV2-ITR
**33**	AAV CMV-driven AcrIIA4-2x*miR-122* (Addgene # 120298)	AAV4-ITR, U6 promoter, *Spy*Cas9-sgRNA-scaffold, CMV promoter, AcrIIA4, 2xmiR-122 binding sites, BGH polyA, AAV2-ITR
**34**	AAV CMV-driven AcrIIA4-2x*miR-1* (Addgene # 120299)	AAV4-ITR, U6 promoter, *Spy*Cas9-sgRNA-scaffold, CMV promoter, AcrIIA4, 2xmiR-1 binding sites, BGH polyA, AAV2-ITR

Supplementary Table S1 continued

**35**	AAV CMV-driven mCherry-AcrIIA4-*scaffold*	AAV4-ITR, CMV promoter, NLS-mCherry-AcrIIA4, scaffold sequence, BGH polyA, AAV2-ITR
**36**	AAV CMV-driven mCherry-AcrIIA4-2x*miR-122*	AAV4-ITR, CMV promoter, NLS-mCherry-AcrIIA4, 2xmiR-122 binding sites, BGH polyA, AAV2-ITR
**37**	AAV CMV-driven AcrIIC1-*scaffold* (Addgene # 120300)	AAV4-ITR, U6 promoter, *Spy*Cas9-sgRNA-scaffold, CMV promoter, AcrIIC1, scaffold sequence, BGH polyA, AAV2-ITR
**38**	AAV CMV-driven AcrIIC3-*scaffold* (Addgene # 120301)	AAV4-ITR, U6 promoter, *Spy*Cas9-sgRNA-scaffold, CMV promoter, AcrIIC3, scaffold sequence, BGH polyA, AAV2-ITR
**39**	AAV CMV-driven AcrIIC1-2x*miR-122* (Addgene # 120302)	AAV4-ITR, U6 promoter, *Spy*Cas9-sgRNA-scaffold, CMV promoter, AcrIIC1, 2xmiR-122 binding sites, BGH polyA, AAV2-ITR
**40**	AAV CMV-driven AcrIIC3-2x*miR-122* (Addgene # 120303)	AAV4-ITR, U6 promoter, *Spy*Cas9-sgRNA-scaffold, CMV promoter, AcrIIC3, 2xmiR-122 binding sites, BGH polyA, AAV2-ITR

MicroRNA Expression

**41**	CMV-miR-122	CMV promoter, miR-122, BGH polyA
**42**	CMV-miR-1	CMV promoter, miR-1, BGH polyA

AAV Production Plasmids

**43**	Adenoviral helper plasmid	Ad2 VA RNA, Ad2 E4, Ad2 E2A
**44**	WHc2	AAV2 cap, AAV2 rep
**45**	WHc6	AAV6 cap, AAV2 rep

Stuffer DNA

**46**	pcDNA3.1$^{(-)}$	empty vector (inert DNA stuffer)

Supplementary Table S2. sgRNA target sites. Sequences are in 5' to 3' direction; the PAM sequence is indicated in bold. References indicate the publications originally reporting the corresponding sgRNAs.

Gene (Reference)	Cas9 orthologue	Target Sequence
Firefly luciferase	*Spy*Cas9	GGACTCTAAGACCGACTACC**AGG**
Tet-responsive element (2)	*Spy*Cas9	TCTCTATCACTGATAGGGAG**TGG**
EMX1 (2)	*Spy*Cas9	GAGTCCGAGCAGAAGAAGAA**GGG**
Rosa-26 (3)	*Spy*Cas9	ACTCCAGTCTTTCTAGAAGA**TGG**
CCR5 (2)	*Spy*Cas9	TGACATCAATTATTATACAT**CGG**
AAVS1 (4)	*Spy*Cas9	GGGCCACTAGGGACAGGAT**TGG**
VEGFA (5)	*Nme*Cas9	GCGGGGAGAAGGCCAGGGGTCACT**CCAGGATT**

Supplementary Table S3. Primers used for genomic PCRs. Sequences are in 5' to 3' direction.

Locus (species)	Direction	Sequence
EMX1 (human)	forward	GGAGCAGCTGGTCAGAGGGG
	reverse	GGGAAGGGGGACACTGGGGA
Rosa-26 (mouse)	forward	CGTGCAAGTTGAGTCCATCCGCC
	reverse	ACTCCGAGGCGGATCACAAGCA
CCR5 (human)	forward	GAGCCAAGCTCTCCATCTAGT
	reverse	GCCCTGTCAAGAGTTGACAC
AAVS1 (human)	forward	TCCAGGGGTCCGAGAGCTCAGCTAG
		CCCCTTACCTCTCTAGTCTGTGC
	reverse	CCAGACAGCCGCGTCAGAGCAGCTC
VEGFA (human)	forward	TCCAGATGGCACATTGTCAG
	reverse	AGGGAGCAGGAAAGTGAGGT

Supplementary References

1. Chew, W.L., Tabebordbar, M., Cheng, J.K., Mali, P., Wu, E.Y., Ng, A.H., Zhu, K., Wagers, A.J. and Church, G.M. (2016) A multifunctional AAV-CRISPR-Cas9 and its host response. *Nat Methods*, **13**, 868-874.

2. Nihongaki, Y., Kawano, F., Nakajima, T. and Sato, M. (2015) Photoactivatable CRISPR-Cas9 for optogenetic genome editing. *Nat Biotechnol*, **33**, 755-760.

3. Chu, V.T., Weber, T., Wefers, B., Wurst, W., Sander, S., Rajewsky, K. and Kuhn, R. (2015) Increasing the efficiency of homology-directed repair for CRISPR-Cas9-induced precise gene editing in mammalian cells. *Nat Biotechnol*, **33**, 543-548.

4. Mali, P., Yang, L., Esvelt, K.M., Aach, J., Guell, M., DiCarlo, J.E., Norville, J.E. and Church, G.M. (2013) RNA-guided human genome engineering via Cas9. *Science*, **339**, 823-826.

5. Amrani, N., Gao, X.D., Liu, P., Edraki, A., Mir, A., Ibraheim, R., Gupta, A., Sasaki, K.E., Wu, T., Donohoue, P.D. *et al.* (2018) NmeCas9 is an intrinsically high-fidelity genome-editing platform. *Genome Biol*, **19**, 214.
